# How Effective Are Mindfulness-Based Interventions for Reducing Stress and Weight? A Systematic Review and Meta-Analysis

**DOI:** 10.3390/ijerph20010446

**Published:** 2022-12-27

**Authors:** Elena Sosa-Cordobés, Juan Diego Ramos-Pichardo, José Luis Sánchez-Ramos, Francisca María García-Padilla, Elia Fernández-Martínez, Almudena Garrido-Fernández

**Affiliations:** Department of Nursing, University of Huelva, 21071 Huelva, Spain

**Keywords:** mindfulness, mindfulness-based intervention, weight loss, stress, psychological, adult

## Abstract

Stress contributes to the development and maintenance of obesity. Mindfulness-based therapies are being used to reduce stress and promote weight reduction and maintenance. This study aimed to determine the efficacy of mindfulness-based interventions for stress and weight reduction in the short, medium, and long term. Searches on PsycINFO, Medline, CINAHL, Scopus, WOS, and Science Direct were conducted until March 2021. Intervention studies with a sample of adults were included; these evaluated a mindfulness-based intervention and used stress and weight or body mass index as outcome variables. These criteria were met by 13 articles. A meta-analysis of 8 of the 13 articles was performed with a random-effects or fixed-effects model, depending on the level of heterogeneity between studies. Mindfulness-based interventions had a small effect on stress reduction over a 3-month period: effect size (standardized mean difference) = −0.29 (95% CI: −0.49, −0.10). However, no significant evidence was found for stress reduction from 3 months onwards, nor for weight or body mass index reduction in any period. Mindfulness-based interventions are effective in reducing stress in the short term, but not in the medium or long term, nor are they effective for weight or body mass index. More robust and longer study designs are needed to determine their effects.

## 1. Introduction

Global obesity has significantly increased since 1975. A sizable portion of the total population lives in nations where overweight, and obesity causes a greater number of deaths than malnutrition. A body mass index (BMI) above the recommended value is a key risk factor for non-communicable diseases such as diabetes, cardiovascular disease, musculoskeletal disorders, and some types of cancers. Overweight and obesity result in approximately 2.8 million deaths every year [1]. 

The source of overweight and obesity is an energy imbalance between calorie intake and calories used [2]. The relationship between emotional state and habits is well known [3]. Emotional eating has been considered as a strategy to mitigate anxiety, stress, sadness, and other negative emotions. Feelings affect food choices extraordinarily, particularly “comfort foods”, which rapidly restock energy levels consumed during the stress reaction, thus causing an immense feeling of well-being for those who eat them [4].

Lazarus (1999) described stress in his transactional model as an association between the person and the circumstances that have been assessed by the individual as being beyond their abilities and resources to cope [5]. Stress can play a key role in developing and maintaining overweight. Cortisol is known to increase the appetite for comfort, energy-dense, and often unhealthy foods. Oddly, in current society, the obesity pandemic concurs with a rise in factors that promote the production of cortisol, such as consumption of high glycemic index foods, chronic stress, and reduced sleeping hours [5,6]. 

The treatment of obesity and its comorbidities is important because most people who lose weight do not successfully maintain the condition [3]. The standard treatment of weight loss depends on two elements: following a healthy, low-calorie diet and performing regular exercise [7]. Nonetheless, it is essential to supplement diet and exercise with interventions aimed at reducing risk factors such as stress [6].

Mindfulness is an idea introduced in the Western world by Jon Kabat-Zinn in 1982 and is defined as “the objective perception of reality in the present without a critical attitude” [8,9]. Mindfulness-based interventions have significantly spread their use in recent years and have been put into practice in numerous health settings. Likewise, higher wellness and personal satisfaction in both healthy and sick people were additionally achieved [10,11]. Kabat-Zinn developed the eight-week mindfulness training course known as MBSR, or mindfulness-based stress reduction, at the University of Massachusetts Medical Centre in 1979. Courses include daily homework, a one-day retreat with a 7 h mindfulness practice, and weekly group meetings that last 2.5 h each (these generally last 45 min). Although the course is challenging, many people feel that their health and wellness advantages make up for the time investment. Group discussions are a crucial component of the curriculum. Patients practice a range of meditative and physical techniques during class to increase their resilience and mindful awareness. These include loving-kindness meditation, sitting meditation, yoga and other types of moderate mindful movement, body scans (a type of meditation practice), and sitting meditation [10]. However, an eight-week mindful eating program will teach patients how to approach food and eating with less anxiety and more comfort [7].

Stress is already presumed to play a relevant role in developing and maintaining obesity. Considering that mindfulness is effective for stress decrease and emotional eating, its application for reducing stress-induced weight appears to be encouraging [12,13,14]. 

One theory regarding the possible connection between mindfulness, stress, and weight is that mindfulness improves stress-handling patterns, so it might add to a decline in the occurrence of overweight and its persistence through time [10,15,16]. Some recent works have confirmed that stress plays a key role in the association between mindfulness, weight, and eating behaviors [10,13,16]. Recent studies have demonstrated the effectiveness of mindfulness-based interventions for stress reduction [17,18,19].

In any case, it is not known whether these interventions can affect weight handling. Likewise, a systematic review was developed to evaluate and synthesize the evidence of the effectiveness of mindfulness-based electronic interventions for weight and stress management [6].

Until now, there have been no reviews that contemplate these two results as our study does, and this is why it is novel. The goal of this systematic review and meta-analysis was to evaluate and integrate proof for the adequacy of mindfulness-based interventions in decreasing stress, weight, and BMI in an adult population.

## 2. Materials and Methods

### 2.1. Search Strategy

A comprehensive search was conducted of worldwide databases based on the proposals of the PRISMA guidelines by Moher et al. (2016) [20]. The search was conducted in October 2020 and there was no limited publication period. The databases of reference were: PsycINFO, Medline, CINAHL, Scopus, WOS, and Science Direct. The search strategy was: (“mindfulness” OR “mindful” OR “mindfulness-based interventions”) AND (“stress” OR “mental stress”) AND (“weight” OR “BMI”). All terms were searched in both regular (title or abstract) and controlled language (thesaurus).

### 2.2. Inclusion and Exclusion Criteria

As this is an incipient study area, a scarcity of mindfulness-based approaches to weight and stress was expected to be found. Hence, the criteria for inclusion were relatively wide to be able to include any mindfulness-based intervention. 

Original articles that met inclusion criteria were incorporated: (i) intervention studies provided by any health professional; (ii) a sample of adults of age; (iii) assessment of mindfulness-based intervention, either as a treatment on its own or within a greater multicomponent intervention; and (iv) stress, weight, and/or BMI as outcome variables. Observational studies and articles that did not measure stress with a validated scale were excluded.

### 2.3. Screening

Firstly, potential articles were selected according to the title and the abstract. After reading the complete text, it was then determined whether the article fitted the selection criteria proposed for the study. The process was performed by two researchers (E.S.-C. and E.F.-M.), independently. If there was disagreement between the two, a third reviewer (F.M.G.-P.) helped to provide a consensus. Abstracts that met the criteria were reviewed in full text. Text articles that met the pre-defined criteria were incorporated into the final study.

### 2.4. Procedure and Data Extraction

Quantitative data were extracted according to the sample size, with measures of outcome variables pre- and post-intervention detailed for the treatment and non-treatment groups.

Two reviewers (E.S.-C. and E.F.-M.) undertook this process, autonomously, to guarantee maximum reliability. A third researcher (F.M.G.-P.) helped to resolve any discrepancies through consensus meetings. 

### 2.5. Risk of Bias Assessment

The Cochrane Collaboration’s instrument for evaluating the risk of bias according to Higgins et al. [21] was utilized. Two researchers (E.S.-C. and F.M.G.-P.) assessed the risk of bias on their own, resolving disagreements in a consensus meeting involving a third researcher [E.F.-M.].

### 2.6. Data Analysis

For the meta-analysis, works that offered arithmetical information for sample size, mean, and standard deviation of outcome variables (stress, weight, and BMI) were selected. Standardized mean differences, 95% confidence intervals, total effect sizes, and weights were calculated for each of the studies.

To evaluate heterogeneity between studies, the I^2^ statistic was calculated and estimated values of 25%, 50%, and 75% were deciphered as low, intermediate, and high heterogeneity, respectively [22]. Meta-analysis was performed using MetaEasy (Version 2013, Manchester, UK): a meta-analysis add-in for Microsoft Excel [23]. When studies evaluate the same result but do so using various methods, the standardized mean difference (effect size) is employed as a summary statistic. In this case, standardizing the study data to a consistent scale is required before they can be integrated. The extent of the intervention effect in each research about the variability seen in that study is expressed by the standardized mean difference [22].

## 3. Results

### 3.1. Selection Process

**Figure 1:**
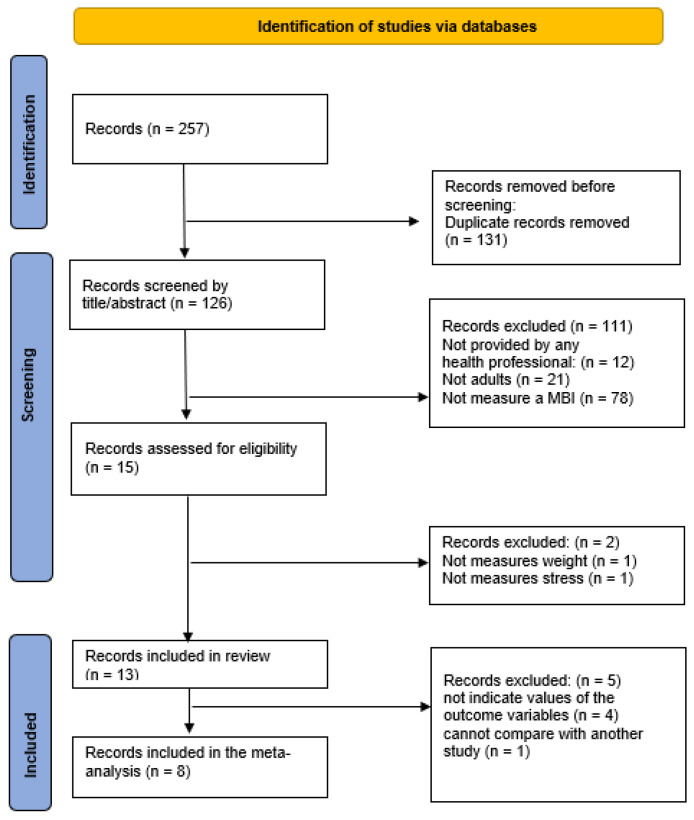
PRISMA flow diagram.

Figure 1 shows the selection process. A total of 257 studies was identified, of which 131 duplicate articles were removed. From the remaining 126 studies, 15 were chosen by title and abstract for their significance to the study question, with 2 studies rejected for two different reasons (see Figure 1). A total of 13 articles met the inclusion criteria and 8 were incorporated into the meta-analysis.

The 13 studies had analogous aims: applying mindfulness-based interventions in adults for stress and weight reduction, among other variables. There were 6 studies with a focal point on testing already existing interventions, whereas the other 6 focused on examining new interventions designed by the authors [15,24,25,26,27,28].

However, in 5 cases, studies took into account that, in addition to stress and weight and/or BMI, other main outcome variables were considered, such as quality of life and body image acceptance [29]; biomarkers of inflammation and metabolism [24]; physiological markers of a chance of having a heart attack [25]; cortisol awakening response in abdominal fat [30]; and emotional eating, telomerase activity, and metabolic variables [31].

Moreover, some other studies considered mindfulness, mindful eating, regular exercise, and eating behaviors [26]; reward-based eating [10]; anxiety and depressive symptoms [32]; fasting glucose and blood pressure [33]; depression, sleep quality, regular exercise, and healthy eating [27]; diabetes biomarkers, body measurements, personal satisfaction, diet, and activity measures [34]; barriers and facilitators of healthful eating behaviors, being active, and practicing mindfulness [28]; and depression [35].

### 3.2. Characteristics of the Included Studies

Table 1 describes the main characteristics of the members and the interventions identified. However, some of the cells in the main outcomes column are missing numerical values of stress reduction, BMI, and weight as outcome variables before and after mindfulness-based interventions due to their absence in the articles. Randomized controlled trials (RCT) and pre-post and mixed methods studies were included for analysis.

Of the 13 studies, 6 used a randomized controlled experimental design [6,10,13,24,32,33]; 2 studies were pilot studies [25,30]; 3 studies used a pre-post design [29]; 1 used a mixed design [36]; and 1 was quasi-experimental [35].

Sample sizes were between 10 and 236 participants, with all aged 18 years and older. Of the 13 studies, 8 included samples of overweight participants [9,10,24,25,28,29,30,37], while the rest allowed normal-weight individuals to be included in the intervention [26,27,32,35,36]. However, 1 study included a sample who had undergone bariatric surgery [24]; another included man with CHD [32]; a third study included subjects with pre-diabetes [36]; and, finally, 1 study selected pregnant women [35]. As for the sample structure by sex, 6 of the articles only incorporated females [9,25,27,30,35,37], 6 other studies included both men and women [6,24,28,29,36,38], and 1 article only included men [32].

The MBSR program was used in 6 of the eligible studies [6,27,30,32,36,37], with 1 study using MBSR together with MB-EAT [30], another study with ME [6], and another with a mobile app [27]. The interventions differed in attributes in terms of the length of sessions and duration of daily exercise. In most of the interventions, the duration of the planning was 8 weeks, ranging from 4 to 24 weeks. Versatility was higher regarding session length and periodicity, as shown in Table 1.

As regards the effects, no decrease in weight levels was identified in 6 of the 13 studies [25,29,32,35,37,38], but 8 of the interventions produced a decrease in stress [10,25,26,27,29,32,35,37]. On the contrary, increased stress was shown by 2 studies [24,28] and, in 1 of them, moreover, this increase was identified in weight [24].

The tool used to assess the outcome variable stress in all the articles was the Perceived Stress Scale, adjusted to the attributes of the sample population, or its abbreviated versions PSS-10 [28] and PSS-4 [37]. Height was measured with a wall-mounted stadiometer and weight was measured to the nearest 0.1 kg using a digital scale, with the participant generally dressed in light clothing. 

Some of the finally eligible works followed a longitudinal design and described follow-up outcomes at 3 months [10,24,25,36], 6 months [10,24,36], 12 months [10], and 18 months [10]. The rest of the studies did not involve a follow-up. The issue of adherence to the plan and frequent attendance at sessions by participants was discussed in some studies. The participants’ opinions on the interventions were not evaluated in the other studies.

### 3.3. Risk of Bias

**Figure 2:**
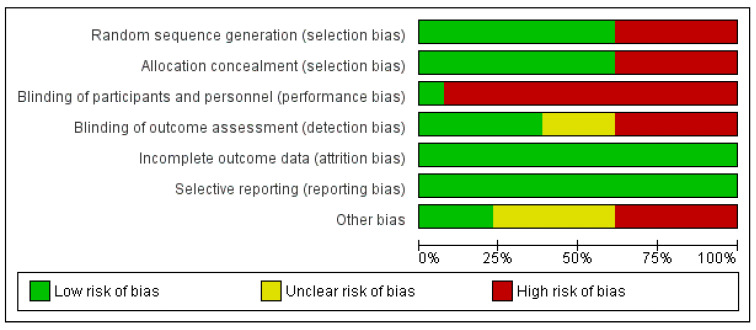
Risk of bias: reviewers’ judgments of each element of risk of bias are presented as percentages across all included studies.

**Figure 3:**
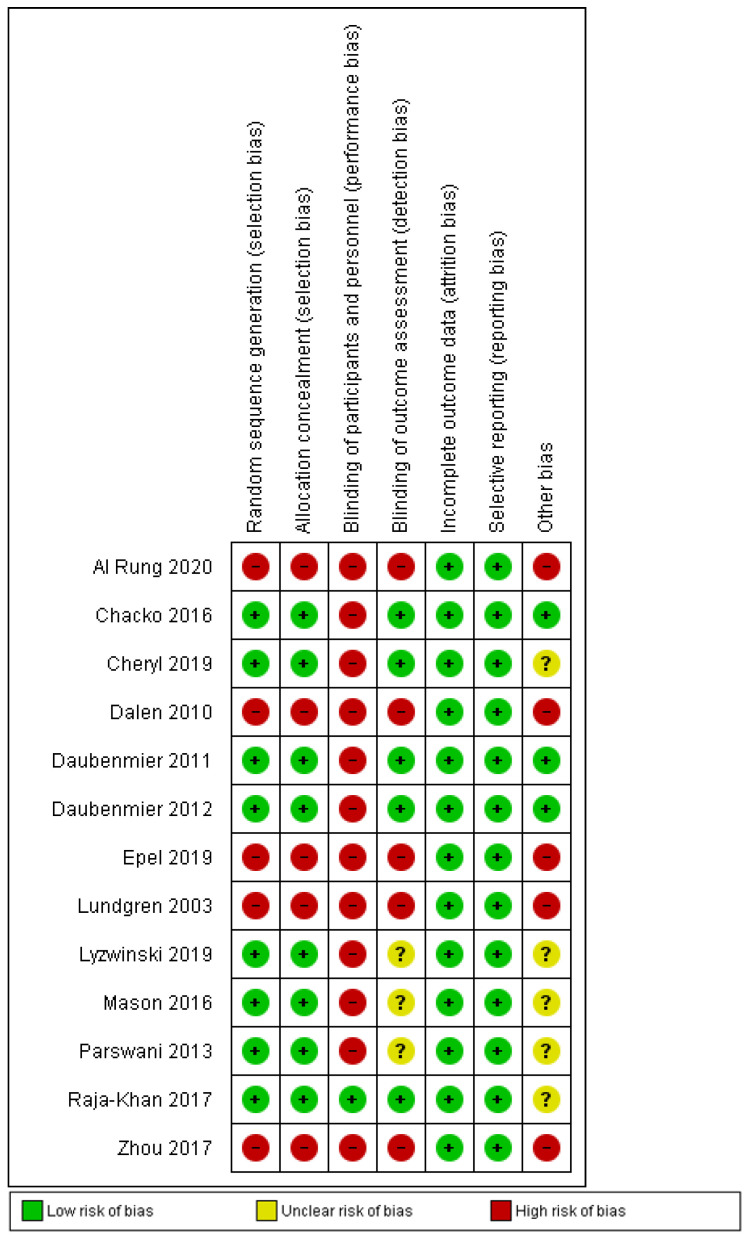
Summary of risk of bias: reviewers’ judgments of each element of risk of bias for each included study ([10,13,24,25,26,27,28,29,30,31,32,33,35]).

The quality of methodological approaches was heterogeneous and numerous works did not offer enough data to appropriately assess the risk of bias. According to the Cochrane Collaboration’s tool for assessing the risk of bias, this was significantly high for performance bias (blinding of participants and staff) and intermediate for the selection and detection of bias domains. Attrition and reporting risk of bias was low according to the reviewers’ judgments. An outline of authors’ opinions on the risk of bias for those studies eventually included is shown in Figure 2; the risk of bias in each study is shown in Figure 3.

### 3.4. Meta-Analysis

**Figure 4:**
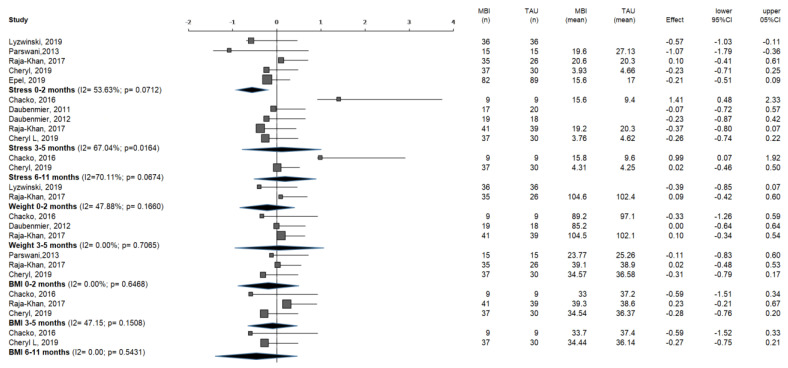
Meta-analysis of mindfulness-based interventions ([10,13,24,26,31,32,33,35]). Horizontal axis represents effect size (standardized mean difference); I2, heterogeneity coefficient; *p*, significance for Q-statistic; MBI, mindfulness-based intervention; TAU, therapy as usual.

The meta-analysis (see Figure 4) was performed on the present study with random effects, except for the studies which measure stress at < 6 months; they were performed with a fixed-effects model. There were 4 studies [25,27,28,29] excluded from the meta-analysis because they did not indicate arithmetic values of the outcome variables in their assessment of the efficacy of the mindfulness-based intervention (MBI), and thus reported incomplete outcomes. Mason et al. (2016) [30] was the only study that measured outcome variables at 12 and 18 months. 

#### 3.4.1. Outcome Variable: Stress

There were 456 participants in 5 of the selected studies, which measured stress during <3 months. In 4 of the studies [26,32,35,36], a non-relevant positive impact of the intervention was observed on the treatment group, compared to the TAU group. A very slight negative non-relevant impact was shown in only 1 of the studies [37]. 

Yet, another 5 of the selected studies, with a total of 215 participants, measured stress at <6 months as an outcome variable. In 4 of the studies, a non-relevant positive impact of the program was observed [9,30,36,37], and only 1 study showed a negative effect [24].

Of the 2 studies, 1 found no effect and the other a significant worsening. The joint estimate showed a non-significant worsening [24,36]. As could be seen in the meta-analysis conducted, when the results of all the studies were integrated, a significant effect on stress reduction was obtained in the immediate effect (<3 months): −0.30 (95% CI: −0.49, −0.10). In the other periods (<6 months and <12 months), this effect was not observed: −0.01 (95%CI: −0.47, 0.45) and 0.23 (95%CI: −0.20, 0.65), respectively. 

#### 3.4.2. Outcome Variable: Weight

Based on the meta-analysis outcomes, MBIs did not prove to be successful in weight reduction. There were 2 studies incorporated for the analysis of the weight variable <3 months, with a total of 143 members. In 1 of the studies [26], a non-significant improvement in weight was noticed, while the other had a very slight non-relevant negative effect [37].

Furthermore, 3 studies were incorporated for the analysis of the weight variable at < 6 months, with a total of 118 members, with 1 of the studies [24] showing a non-relevant positive effect of the plan on the treatment group, contrasted with the non-treatment group. 

The others showed a non-significant null or negative effect [15,24]. Based on the meta-analysis undertaken, an impact of the intervention on weight cannot be established as the impact size is −0.17 (95%CI: −0.51, 0.17) at <3 months and −0.01 (95%CI: −0.32, 0.35) for <6 months.

#### 3.4.3. Outcome Variable: BMI

As happened with the weight outcome variable, MBIs do not beneficially affect the BMI outcome variable. There were 3 studies incorporated for the study of BMI estimated at <3 months, with a total of 151 members. Of these, 2 studies [32,36] showed a non-relevant positive effect of the program on BMI, while the third showed a non-relevant negative effect [37].

Before 6 months, 3 research works were assessed, with a total of 139 participants. Of these, 2 works [24,36] showed a non-relevant weight reduction, while the third showed a non-relevant increase [37]. Lastly, the estimation of BMI at <12 months was evaluated in 2 studies, with a total of 86 participants. In both studies [24,36], a non-relevant impact of the program was seen in the treatment group when contrasted with the control group. 

As indicated by the meta-analysis performed, an impact of the intervention on BMI does not exist because the effect size for <3 months is −0.14 (95%CI: −0.46, 0.17); for <6 months, it is −0.07 (95%CI: −0.37, 0.24); and for <12 months, it is −0.34 (95%CI: −0.76, 0.09). 

## 4. Discussion

This systematic review analyzed 13 articles that assessed the viability of mindfulness-based interventions in reducing adults’ stress and weight. The outcomes provide proof that these interventions are effective in reducing stress in the short term, yet not in the medium or long term, nor for weight or BMI. In addition, the risk of bias makes confirming the validity of these results impossible. There exists great variability in terms of the application and handling of the interventions, with the effect varied depending on the amount of daily meditation practice, among other variables. 

This is significant, as chronic health issues, especially those related to obesity, contribute to the suffering and ill health of a very large portion of the population. Mindfulness-based interventions have proven to be beneficial for short-term stress handling and for reducing stress in different populations, as the accessible literature shows. 

This is the second systematic review and first meta-analysis to assess the effectiveness of these interventions on adult populations, and it has as an added novelty that we have assessed weight reduction, in the short, medium, and long term [6].

It was identified that 6 studies applied the MBSR program to adult populations, yet with certain heterogeneity as regards the implementation of the program according to their duration or intervals between the different sessions. Of the 13 studies selected, 5 offered incomplete information on their outcomes. Therefore, only 8 could be eventually considered for the meta-analysis. 

Regarding the stress variable, a small effect size was found. Moreover, the data show that mindfulness programs can be effective in stress reduction in the short term. In addition, the confidence intervals were relatively tight, i.e., with the lower limits close to 0 (zero effect). Taking this into consideration, the effectiveness of mindfulness-based interventions in reducing stress has been stated as relative. The use of mindfulness interventions has been recently debated in different research [39,40,41]. The present meta-analysis offers data that indicate scarcely relevant effects on reducing stress. This also applies to other systematic reviews [6,42]. However, the effect is very limited over time, which would provide a basis for discussing the persistence of the intervention, adherence in the medium/long term, and the need for participants to incorporate meditation habits and routines. Moreover, it is also convenient to suggest new studies that have longer-term interventions planned.

Overall, mindfulness-based interventions need a high level of adherence and commitment from the people who participate in them to obtain acceptable outcomes; they demand daily practice, which can imply a great effort [43]. When the participant is committed to practicing meditation, more highly successful results are obtained. Indeed, the present assessment of stress and weight variables has shown that regularity and adherence to meditation practice are a key for the effectiveness of the intervention [26,28].

There was a high or ambiguous risk of bias in all cases except for attrition and reporting. There was no article with a low risk of bias in all the analyzed domains; thus, the quality of the data offered by the studies appears to be somewhat uncertain. It may very well be inferred that the positive outcomes introduced by the chosen studies are not substantial. In any case, the negative evaluation of the risk of misleading results is thought to be impacted by a non-ideal use of the Cochrane Collaboration tool for assessing the risk of bias in this kind of study. Mindfulness-based interventions need participation in group sessions; however, it is also mandatory for the interventions to be successful to have the necessary motivation to perform the daily activities between sessions as an individual task, and the variables usually analyzed are self-reported. In this manner, randomization, and blinding processes, such as those normally conducted in studies of different interventions, are not possible. Mindfulness-based interventions have also been reported with a generally unclear risk of bias by other researchers [19,26]. In search of an accurate assessment of the risk of misleading results in the interpretation, fostering an expansion or change of the Cochrane Collaboration’s tool for assessing the risk of bias that is better adjusted to this kind of non-pharmacological intervention is regarded as essential.

One of the strengths of this study is the fact that six databases were searched for all types of mindfulness-based interventions, and that both stress and weight were analyzed for effectiveness. In addition, a novel area of research was identified, and this needs more in-depth study and new research choices. While databases were systematically searched, there is always a possibility that some studies could have been neglected.

This review also has limitations. For instance, the search strategy was directed to international databases (PsycINFO, Medline, CINAHL, Scopus, WOS, and Science Direct); therefore, articles published by other sources or in the grey literature could have been disregarded. 

Future lines of research ought to dig further into these two ideas that affect weight reduction, with intervention approaches specifically developed for appropriately avoiding any risk of misleading results in the interpretation phase, considering the attributes of mindfulness-based interventions. Furthermore, it would be fitting to consider other outside factors connected with weight gain. To this end, performing mindfulness-based interventions in well-being and dietary facilities, and conducting the studies in circumstances where daily meditation and participants’ commitment are encouraged, would be optimal. These approaches would help to better assess the effectiveness of intervention programs with similar lines of action.

There is no evidence about the effectiveness of mindfulness-based interventions for weight reduction. More research with higher methodological quality and adequate power (sample size) is required to assess the clinical utility of these interventions for stress and weight reduction, together, in the short, medium, and long term.

## 5. Conclusions

This systematic review analyzed 13 articles that assessed the effectiveness of mindfulness-based interventions in reducing adults’ stress and weight. These interventions are effective in reducing stress in the short term, yet not in the medium or long term, nor are they effective for weight or BMI. In addition, the risk of bias makes confirming the validity of these results difficult. There exists great variability in terms of the application and handling of the interventions, and the effect is varied depending on the amount of daily meditation practice, among other variables.

Mindfulness-based interventions seem to be effective for stress reduction in the short term, though they do not seem to be effective for weight reduction. However, because of the few studies found, conclusions about their general effectiveness cannot be delineated.

## Figures and Tables

**Table 1:** Characteristics of the included studies.

Authors	Sample	Methods	Location	Aim	Intervention	Control	Duration	Variables	Main outcomes
Lundgren et al., 2003 [29]	N = 19 Mean age = 44.8	Pre-post	USA	Assess whether IGs decrease general symptomatology	Mindfulness meditation practice + traditional behavioral treatment	No control group	20 weeks	General symptomatology, stress, quality of life, acceptance of body image, percent weight loss, and binge eating	PSS pre-post (*p* > 0.05). BMI not significant pre-post measures differences. Several completed homework was positively correlated with the amount of weight lost (r = 0.55, *p* = 0.0018). Number of meditations and binge eating score (r = 0.46, *p* = 0.046).
Chacko et al., 2016 [24]	N = 18 IG = 9 CG = 9 Mean age = 54	RCT	USA	Assess a new MBI aimed at weight management after a bariatric medical procedure	Mindfulness with modified versions of traditional behavioral plan	1 h personalized advice session with a dietitian	10 weeks	Viability and acceptance of MBI, weight, eating behaviors, and psychological results	No differences in weight, BMI, or circumference. At 12 weeks, higher scores on perceived stress and depression (*p* < 0.05).
Dalen et al., 2010 [25]	N = 10 Mean age = 44	Pilot study: pre-post	Mexico	Mindful eating and living	Training in mindfulness meditation, mindful eating, and group discussion	No control group	6 weeks	Changes in weight, BMI, eating behaviors, and psychological distress.	All participants significantly lost weight (*p* < 0.001) Perceived stress (after 12 weeks) (*p* = 0.02).
Daubenmier et al., 2011 [10]	N = 47 IG = 24 CG = 23 Mean age = 32.5	Randomized waitlist-controlled pilot study	USA	Assess the impact of a mindfulness intervention on stomach adiposity in women who suffer from overweight or obesity	Included nine 2.5 h sessions and one 7 h silent day of coordinated reflection practice following class 6	Waitlist group	4 months	Mindfulness, eating behaviors, mental misery, weight, and cortisol arousing reaction stomach fat	No difference was found between groups regarding mean weight. The effect size was medium for self-perceived levels of stress.
Daubenmier et al., 2012 [13]	N = 47 IG = 24 CG = 23 Mean age = 32.5	RCT	USA	Assess MBIs whether decreased psychological distress, eating patterns, and metabolic characteristics	MBSR and MB-EAT	Waitlist group 2 h diet and exercise information	4 months	Stress eating, telomerase activity, psychological distress, eating behaviors, and metabolic characteristics	Not a significant change after some time in the levels of self-perceived stress; both groups maintained their weight over time.
Lyzwinski et al., 2019 [26]	N = 90 IG= 45 CG = 45 Mean age = 20.16	RCT	Australia	Assess a mindfulness app for weight, weight-related conduct, and stress management	Intervention group was given the mindfulness app with ME and MBSR strategies	Behavioral self-observation; electronic diary for diet and physical exercise	11 weeks	Weight, stress, mindfulness, mindful eating, physical exercise, and eating behaviors	There were no statistically significant differences in weight between both the mindfulness app intervention and control e-diary groups at follow-up using ANCOVA (*p* = 0.27). Pairwise comparisons indicate that the control had stress levels that were 3.921 points higher on the PSS than the IG (*p* < 0.05; 95% CI 0.591–0.592).
Mason et al., 2016 [30]	N = 194 IG = 100 CG = 94 Mean age = 47	RCT	USA	Assess post-intervention reward-driven eating and psychological stress acting as an intermediary of the effect of the intervention arm on weight loss at 12 and 18 months	Nutritional and physical exercise plan with mindfulness training.	Diet and exercise intervention and active control	5.5 months	Reward-based eating, psychological stress, and weight	The whole sample significantly lost weight and self-perceived stress decreased after 12 months.
Parswani et al., 2013 [31]	N = 30 IG = 15 CG = 15 Mean age = 47.27	RCT	India	Assess the impact of the MBSR program on health symptoms	MBSR	TAU = health education session	MBSR = 8 weeks TAU = 1 session	Anxiety and depressive symptoms, and self-perceived stress	A significant reduction was observed in symptoms of anxiety and depression, perceived stress, BP, and BMI in patients in the MBSR group after the completion of the intervention assessment. At 3-months follow-up, therapeutic gains were maintained in patients of the MBSR group.
Raja-Khan et al., 2017 [32]	N = 86 IG = 42 CG = 44 Mean age = 44.5	RCT	USA	Evaluate MBSR on women who suffer from overweight or obesity	MBSR	Health education and stress management incorporated to minimize bias of subject expectations	8 weeks	Mindfulness, perceived stress, fasting glucose, and blood pressure	Compared to health education, the MBSR group demonstrated significantly improved mindfulness at 8 weeks (mean change from baseline, 4.5 vs. −1.0; *p* = 0.03) and significantly decreased perceived stress at 16 weeks (−3.6 vs. −1.3, *p* = 0.01). In the MBSR group, there were significant reductions in fasting glucose at 8 weeks (−8.9 mg/dL, *p* = 0.02) and 16 weeks (−9.3 mg/dL, *p* = 0.02) compared to baseline.
Rung et al., 2020 [27]	N = 236 Mean age = 46.1	Pre-post	USA	Evaluate the viability and acceptance of a mobile mindfulness app in real daily-life conditions in a pilot study	Mobile MBSR training program: Headspace	No control group	30 days	Viability and acceptance of the app and characteristics of app usage, mindfulness, depression, self-perceived stress, sleep quality, physical exercise, BMI, and healthy eating	Compared to health education, the MBSR group showed significantly lower levels of perceived stress at 16 weeks, compared to previous stages. No significant changes were found in weight in the MBSR group.
Cheryl et al., 2019 [33]	N = 68 IG = 38 CG = 30 Mean age = 52.57	Mixed	USA	Assess MPD to reduce the risk of having diabetes via reduced stress levels	MBSR adapted for prediabetes risk reduction	Conventional diabetes risk-reduction treatment	8 weeks	Diabetes biomarkers, body composition measurements, self-perceived stress, quality of life, and diet and physical exercise measures	Only the MPD group experienced significant reductions in BMI at 3 months. In addition, the MPD group experienced significant reductions in perceived stress at 3 months follow-up, whereas there were no significant changes in perceived stress in the CPD group.
Zhou et al., 2017 [28]	N = 34 Mean age = 56.1	Pre-post	USA	Assess the viability of a multiple-component lifestyle plan	Individual and group advice on nutrition, exercise, and mindfulness	No control group	12 weeks	Barriers and facilitators of healthy eating, being physically active, and practicing mindfulness. Psychological, dietary, physical exercise and anthropometric data, and clinical data	After 12 weeks, questionnaire-derived PSS scores slightly decreased (from 13.7 ± 1.4 to 12.4 ± 1.5, not significant). After 12 weeks’ intervention, body fat% was reduced among males (33.8 ± 2.6 to 28 ± 2.6, *p* = 0.043).
Epel et al., 2019 [35]	N = 225 IG = 115 CG = 105 Mean age = 28	Quasi-experimental study Non-randomized control group	USA	Assess MMT on self-perceived stress, eating behaviors, and gestational weight gain	MMT + prenatal medical care	TAU = prenatal medical mindfulness	8 weeks	Weight gain, self-perceived stress, and depression	The intervention group showed significant decreases from baseline to the post-intervention period in distress (perceived stress (*p* = 0.04) and depression (*p* = 0.007)). There were also improvements in the acceptance of negative experiences (0.006).

MBWL, mindfulness-based weight loss; IG, intervention group; CG, control group; RCT, randomized controlled trial; MBI, mindfulness-based intervention; PSS: perceived stress scale; BMI, body mass index; MBSR, mindfulness-based stress reduction; MB-EAT, mindfulness-based eating awareness training; ME, mindful eating; ANCOVA: analysis of covariance; BP, blood pressure; TAU, treatment as usual; MPD, mindfulness-based diabetes risk-reduction education program for prediabetes; CPD, conventional diabetes risk-reduction education program for prediabetes; MMT, mindful moms training.

## Data Availability

The datasets used and/or analyzed during the current study are available from the corresponding author upon reasonable request.
